# Early detection of breast cancer: the utilization of mammography in Germany

**DOI:** 10.17886/RKI-GBE-2017-125

**Published:** 2017-12-13

**Authors:** Anne Starker, Klaus Kraywinkel, Ronny Kuhnert

**Affiliations:** Robert Koch Institute, Department of Epidemiology and Health Monitoring, Berlin

**Keywords:** BREAST CANCER, MAMMOGRAPHY, CANCER SCREENING, HEALTH MONITORING, GERMANY

## Abstract

Breast cancer is the most common cancer in women in Germany. In addition to physical examinations (palpation) mammographies are offered every two years to women aged 50 to 69 years as part of the mammography screening programme. For this purpose women are invited in writing and recive a leaflet with information about the background, aims procedures, benefits and possible disadvantages that the screening programme might have. The invitation and the leaflet were revised in 2015 to better support the invited women in making an informed decision about whether they should participate in the programme. In the GEDA 2014/2015-EHIS study, the respondents provided the date of their last mammography and the reason for this. A mammography has been performed on 74.2% of women aged 50 to 69 years within the last two years. 80.7% of these women stated that the reason for this was an invitation sent out by the national screening programme.

## Introduction

Breast cancer is the most common cancer in women in Germany. One in eight women suffers from breast cancer at some point, with the mean age of onset being 64 [1]. Breast cancer screening aims to identify breast cancer as early as possible so as to provide a milder and better form of treatment, and, in turn, improve the quality of life of breast cancer patients and their chances of recovery. However, screening cannot prevent the development of breast cancer, and the procedures used to test for the condition may also come with disadvantages and risks.

From the age of 30, women with German statutory health insurance are entitled to an annual breast cancer examination (palpation of the breasts and an examination of the regional lymph nodes) by a gynaecologist. The aim of this examination is to detect palpable changes (nodes) that should clarified diagnostically by imaging techniques such as mammography or ultrasound and, if necessary, by a biopsy. Alongside this examination, women aged 50 to 69 years can have an X-ray of the breast (a mammography) conducted every two years as part of the breast cancer screening programme [2]. The women of this age group are invited to the screening programme on the basis of data from the Residents' Registration Office in writing. A leaflet is also sent out at the same time which provides information about the background, goals, content and the procedure itself as well as any benefits and possible disadvantages that the screening programme might have. The programme is aimed at detecting and treating breast cancer as early as possible and reducing the mortality rate of breast cancer at the population level. One disadvantage of the mammography screening programme is the possibility of overdiagnosis. Overdiagnosis refers to the discovery of tumours that would have either been identified at a later date, or that would have remained undetected and would have caused no negative impact on the person’s quality of life.

**GEDA 2014/2015-EHIS**

**Data holder:** Robert Koch Institute

**Aims:** To provide reliable information about the population’s health status, health-related behaviour and health care in Germany, with the possibility of a European comparison

**Method:** Questionnaires completed on paper or online

**Population:** People aged 18 years and above with permanent residency in Germany

**Sampling:** Registry office sample; randomly selected individuals from 301 communities in Germany were invited to participate

**Participants:** 24,016 people (13,144 women; 10,872 men)

**Response rate:** 26.9%

**Study period:** November 2014 - July 2015

**Data protection:** This study was undertaken in strict accordance with the data protection regulations set out in the German Federal Data Protection Act and was approved by the German Federal Commissioner for Data Protection and Freedom of Information. Participation in the study was voluntary. The participants were fully informed about the study’s aims and content and about data protection. All participants provided written informed consent.

More information in German is available at www.geda-studie.de

Mammographies are also performed outside the screening programme. There are medical reasons for examination, e.g. due to a conspicuous palpation. In addition, opportunistic screenings are also conducted. These are conducted when a doctor recommends a mammography, because of the presence of another illness or an increased risk of breast cancer in the family. No systematic invitations or quality assurance exist in these cases.

In 2013, 71,640 women were newly diagnosed with breast cancer and 17,853 women died from it [3]. From 2005 onwards, there was an increase in disease rates due to the introduction of the mammography screening programme (and the discovery of tumours that would otherwise have been diagnosed later without screening programme). This has been declining since 2009. In addition, the number of detected breast cancer cases at an advanced stage is also declining. In the group of women aged 50 to 69 years more breast cancer cases in their early stages are discovered, which are better to treat and have better chance of recovery. This is one indication that the screening programme is being successful. The main goal of the mammography screening programme is to reduce breast cancer mortality. Since the mid-1990s, i.e. before the programme was introduced, breast cancer mortality in woman aged 50 to 69 years has been falling, mainly due to advances in therapy [3]. The extent to which the mammography screening programme can further reduce breast cancer mortality is not yet predictable due to its short duration.

## Indicator

The GEDA 2014/2015-EHIS study collected data on the utilization of the latest mammography using a self-administered questionnaire which the respondents completed on paper or online. It included the question, ‘When was the last time you had a mammography?’ The following answers were accepted: ‘Within the past 12 months’, ‘1 to less than 2 years ago’, ‘2 to less than 3 years, ‘3 years or more’ and ‘never’. This was followed by the question: ‘What was the reason the last mammography were performed?’ The following answers were accepted: ‘Due to an invitation from the national screening programme’ and ‘Other reasons, such as referral by a gynaecologist’.

This Fact sheet evaluates the utilization of the latest mammography (performed within the last two years) for women aged 50 to 69 years. The response categories ‘Within the last 12 months’ and ‘Between 1 and less than 2 years ago’ were grouped to the category ‘Within the last two years’ because women aged 50 to 69 years are invited every two years to take part in the screening programme for mammography [2]. This allows assessing whether the last mammography was performed within the recommended examination interval. In order to see whether the mammography was performed as part of the national screening programme or for other reasons (see above), the reason for this examination was also assessed. The results are stratified according to age and educational level. A statistically significant difference between groups is assumed if confidence intervals do not overlap.

The analyses are based on data from women aged 50 to 69 years with valid data at the time of the latest mammography (n=4,301). The reason for the last mammography is shown for women who have had a mammogram within the last two years (n=3,143). All calculations were carried out using a weighting factor that corrects for deviations within the sample from the German population (as of 31 December 2014) with regard to age, district type and level of education. The district type reflects the degree of urbanisation and accounts for the regional distribution in Germany. The International Standard Classification of Education (ISCED) was used to classify the participants’ educational and occupational qualifications [4]. A detailed description of the methodology of GEDA 2014/2015-EHIS can be found in Lange et al. 2017 [5] as well as in the article German Health Update: New data for Germany and Europe in issue 1/2017 of the Journal of Health Monitoring.

## Results and discussion

According to GEDA 2014/2015-EHIS data, 74.2% of women aged 50 to 69 years report that they have had a mammogram within the last two years (Table 1). This proportion is smaller in the group of 50- to 54-year-olds than in the higher age groups. Other studies have shown that the use of (cancer) screening varies according to level of educational [6]. Within the individual age groups there are no significant differences in utilization of mammography in terms of education. Nevertheless, women with medium level of education were more likely to have had more frequent a mammography in the last two years than women with low or high level of education.

The majority of women who have had a mammography within the last two years (80.7%) indicated that was undertaken due to an invitation sent out by the national screening programme (Table 2). In the group of women aged 50 to 54 years, this reason is given less frequently than in other age groups. In fact, almost a quarter of women aged 50 to 54 years stated that their last mammography had been conducted for ‘Other reasons’.

A fundamental limitation of the question used in GEDA 2014/2015-EHIS is that the study only asked women why they had undertaken their latest mammography. As such, it is unclear whether women who answered with ‘Other reasons’ had already participated in the screening programme earlier, but have since undergone another mammography outside the screening, for example to clarify a conspicuous palpation. Therefore, although the GEDA 2014/2015-EHIS data suggests that the mammography screening programme reaches many women, mammographies outside the programme remains important.

These results can be classified using data from the German Health Interview and Examination Survey for Adults (DEGS1, 2008-2011) and the results of the annual evaluation of the mammography screening programme implemented by the Kooperationsgemeinschaft Mammographie (German Mammography Cooperation Association).

Compared to data from DEGS1, the utilization of mammography has not increased significantly. For DEGS1, 71.3% of women aged 50 to 69 years reported that they had had a mammography within the last two years [7]; older women slightly more frequently than younger women. In DEGS1, the reason for the last mammography was also asked, but it was possible to specify several reasons (multiple answers). 65.4% of women aged 50 to 69 years who had had a mammography performed within the last two years stated that it had been conducted due to an invitation sent out by the screening programme. Although the results from DEGS1 are only partly comparable with those of GEDA 2014/2015-EHIS (as DEGS1 enabled respondents to provide multiple responses), the data suggest increased participation in the mammography screening programme.

In comparison with the results of other countries participating in the European Health Interview Survey (EHIS), mammography in Germany has been reported slightly more frequently in the last two years than the EU average (73.5% versus 68.8% for 28 countries) [8]. However, in some countries that have also established a mammography screening programme, the proportion was significantly higher (approximately 90%), but the overall range between the countries participating in EHIS [9] remains very high (51.4 percentage points).

The annual evaluation report on the mammography screening programme assesses the participation of women invited to the screening; as such, its results are not comparable with those from DEGS1 and GEDA 2014/2015-EHIS. Since the introduction of the programme, increasing participation in the programme has been reported, whereas a slight decline has been noted for the first time in 2014. The participation rate for all women who were invited to the programme was 54.2% [10] in 2014, which is lower than the EU guidelines’ target participation rate of at least 70% [11]. As such, a relevant section of the eligible women does not take the opportunity of screening; this, of course, demonstrates the opportunity for increased participation in the programme.

As with all screening tests, mammography for early detection of cancer can be associated not only with advantages but also with disadvantages. Overdiagnosis or false positives (suspected cases that lead to a negative result during a follow-up) mean that screening can cause unnecessary stress and needless diagnostic procedures. It can therefore be difficult for women who are invited to screenings to understand the personal benefits of mammography screening. In July 2015, the Federal Joint Committee adopted an amendment of the cancer screening guidelines, which included changing the invitation letter and the leaflet to better assist women in making an informed decision about participating in the mammography screening programme. The changes have been in force since 2016.

## Key statements

- 74.2% of women aged 50 to 69 years have undergone a mammography in the last two years.
- Women aged 50 to 54 years report less frequently than older women that they have undergone a mammography in the last two years.
- As a reason for the last mammography within the last two years, 80.7% of women stated due to an invitation sent out by the national screening programme.

## Figures and Tables

**Table 1:** The utilization of the latest mammography within the last two years, irrespective of the reason for the examination among women aged 50 to 69 years according to age and educational level (n=4,301) Source: GEDA 2014/2015-EHIS

Women	%	(95% CI)
**Women (total)**	**74.2**	**(72.4-76.0)**
**50-54 Years**	**68.5**	**(65.5-71.4)**
Low education	67.7	(59.7-74.8)
Medium education	69.6	(65.7-73.2)
High education	66.1	(61.0-70.8)
**55-59 Years**	**77.4**	**(74.3-80.3)**
Low education	75.1	(66.9-81.9)
Medium education	79.4	(75.0-83.2)
High education	72.9	(67.2-78.0)
**60-64 Years**	**77.8**	**(74.5-80.8)**
Low education	73.3	(65.8-79.6)
Medium education	80.3	(76.1-83.9)
High education	74.8	(68.0-80.7)
**65-69 Years**	**75.1**	**(71.3-78.5)**
Low education	72.7	(65.2-79.1)
Medium education	76.4	(71.2-80.9)
High education	73.8	(66.0-80.3)

CI=confidence interval

**Table 2:** The reason for the latest mammography within the last two years among women aged 50 to 69 years according to age and educational level (n=3,143) Source: GEDA 2014/2015-EHIS

Women	Invitation by the national screening programme		Other reasons	
	%	(95% CI)	%	(95% CI)
**Women (total)**	**80.7**	**(79.0-82.4)**	**19.3**	**(17.6-21.0)**
**50-54 Years**	**77.5**	**(74.3-80.5)**	**22.5**	**(19.5-25.7)**
Low education	83.5	(75.4-89.3)	16.5	(10.7-24.6)
Medium education	76.9	(72.6-80.8)	23.1	(19.2-27.4)
High education	74.5	(68.7-79.6)	25.5	(20.4-31.3)
**55-59 Years**	**84.6**	**(81.5-87.3)**	**15.4**	**(12.7-18.5)**
Low education	82.2	(73.2-88.6)	17.8	(11.4-26.8)
Medium education	87.0	(83.1-90.0)	13.0	(10.0-16.9)
High education	78.2	(72.2-83.2)	21.8	(16.8-27.8)
**60-64 Years**	**80.3**	**(76.7-83.4)**	**19.7**	**(16.6-23.3)**
Low education	82.0	(73.0-88.4)	18.0	(11.6-27.0)
Medium education	82.5	(77.7-86.4)	17.5	(13.6-22.3)
High education	70.1	(61.6-77.4)	29.9	(22.6-38.4)
**65-69 Years**	**80.8**	**(77.0-84.2)**	**19.2**	**(15.8-23.0)**
Low education	75.5	(66.3-82.9)	24.5	(17.1-33.7)
Medium education	84.1	(79.0-88.2)	15.9	(11.8-21.0)
High education	76.6	(67.7-83.6)	23.4	(16.4-32.3)

CI=confidence interval

## References

[1] Robert Koch Institute (Ed) and the Association of Population-based Cancer Registries in Germany (ed) (2016) Cancer in Germany 2011/2012. 10th edition. RKI, Berlin http://www.krebsdaten.de/Krebs/EN/Content/Publications/Can-cer_in_Germany/cancer_chapters_2011_2012/cancer_germa-ny_2011_2012.pdf?__blob=publicationFile (As at: 13.10.2017)

[2] Gemeinsamer Bundesausschuss (2017) Richtlinie über die Früherkennung von Krebserkrankungen. https://www.g-ba.de/downloads/62-492-1461/KFE-RL_2017-07-20_iK-2017-11-08.pdf (As at 13.10.2017)

[3] Robert Koch-Institut (Ed) (2016) Bericht zum Krebsgeschehen in Deutschland 2016. RKI, Berlin http://edoc.rki.de/documents/rki_fv/renGkGzAqwKc2/PDF/28oaKVmif0wDk.pdf (As at 13.10.2017)

[4] Statistical Office of the European Union (Eurostat) (2016) International standard classification of education (ISCED). http://ec.europa.eu/eurostat/statistics-explained/index.php/Glossary:International_standard_classification_of_educa-tion_%28ISCED%29 (As at 13.10.2017)

[5] Lange C Finger JD Allen J (2017) Implementation of the European health interview survey (EHIS) into the German health update (GEDA). Archives of Public Health 75(1):40 28936356 10.1186/s13690-017-0208-6 PMC5603169

[6] Robert Koch-Institut (Ed) (2017) Gesundheitliche Ungleichheit in verschiedenen Lebensphasen. Gesundheitsberichterstattung des Bundes Gemeinsam getragen von RKI und Destatis. RKI, Berlin http://edoc.rki.de/documents/rki_fv/releGa5LqOxGE/PDF/25xI-YiGiDQ6x2w.pdf (As at 13.10.2017)

[7] Starker A Saß AC (2013) Participation in cancer screening programmes. Results of the German Health Interview and Examination Survey for Adults (DEGS1). Bundesgesundheitsbl Gesundheitsforsch Gesundheitsschutz 56(5-6):858-867 http://edoc.rki.de/oa/articles/reGtNNyWhvpc/PDF/27T4csOo-v0aM.pdf (As at 13.10.2017) 10.1007/s00103-012-1655-4 23703507

[8] Statistical Office of the European Union (Eurostat) (2017) Self-reported last breast examination by X-ray among women by age and educational attainment level. http://ec.europa.eu/eurostat/web/health/health-care/data/data-base# (As at 11.08.2017)

[9] International Agency for Research on Cancer (2017) Cancer Screening in the European Union. Report on the implementation of the Council Recommendation on cancer screening. IARC, Lyon

[10] Malek D Kääb-Sanyal V (2016) Jahresbericht Evaluation 2014. Deutsches Mammographie-Screening-Programm. Kooperationsgemeinschaft Mammographie, Berlin

[11] Perry N Broeders M de Wolf C (Eds) (2006) European guidelines for quality assurance in breast cancer screening and diagnosis. Fourth Edition. European Communities, Luxembourg 10.1093/annonc/mdm481 18024988

